# Spinal Cord Stimulation for Refractory Angina Pectoris: Current Status and Future Perspectives, a Narrative Review

**DOI:** 10.3390/jcdd12010033

**Published:** 2025-01-20

**Authors:** Roberto Gazzeri, Jacopo Mosca, Felice Occhigrossi, Marco Mercieri, Marcelo Galarza, Matteo Luigi Giuseppe Leoni

**Affiliations:** 1 Interventional and Surgical Pain Management Unit, San Giovanni–Addolorata Hospital, 00184 Rome, Italy; 2 Department of Anesthesiology, Critical Care and Pain Medicine, La Sapienza University of Rome, 00185 Rome, Italy; 3 Department of Medical and Surgical Sciences and Translational Medicine, Sapienza University of Rome, 00185 Rome, Italy; 4 Regional Service of Neurosurgery, “Virgen de la Arrixaca” University Hospital, 30120 Murcia, Spain

**Keywords:** refractory angina pectoris, spinal cord stimulation, neurostimulation, myocardial ischemia, quality of life, angina pectoris, closed loop, high frequency, dtm, tonic stimulation, burst

## Abstract

Refractory angina pectoris (RAP) is a clinical syndrome characterized by persistent chest pain caused by myocardial ischemia that is unresponsive to optimal pharmacological therapy and revascularization procedures. Spinal cord stimulation (SCS) has emerged as a promising therapeutic option for managing RAP, offering significant symptom relief and improved quality of life. A systematic literature review was conducted to evaluate the clinical effectiveness, mechanisms of action, and safety profile of SCS in treating RAP. Comprehensive searches were performed in PubMed, Scopus, and Web of Science for studies published between 1990 and 2023. Of 328 articles identified, 6 met the inclusion and exclusion criteria for final analysis. The included studies consistently demonstrated that SCS significantly reduces the frequency of anginal episodes and nitroglycerin use while improving exercise capacity and quality of life. Proposed mechanisms include modulation of pain signals via the gate control theory, enhancement of autonomic balance, and redistribution of myocardial perfusion. Novel stimulation modalities, including high-frequency, Burst, and Differential Target Multiplexed (DTM), show potential advantages in enhancing patient comfort and clinical outcomes. Nevertheless, long-term studies are necessary to validate these findings and establish the comparative efficacy of these advanced technologies. SCS is a safe and effective therapy for patients with RAP who are unsuitable for surgical interventions. Innovations in neurostimulation, including closed-loop systems and personalized treatment strategies have the potential to further optimize outcomes. Rigorous clinical trials are needed to consolidate the role of SCS as a cornerstone therapy for the management of RAP.

## 1. Introduction

Refractory angina pectoris (RAP) is a complex clinical syndrome characterized by persistent chest pain due to myocardial ischaemia that is unresponsive to optimal pharmacological treatments or conventional revascularization procedures [1]. This condition is typically observed in patients with advanced coronary artery disease for whom traditional treatment options are either ineffective or contraindicated due to high surgical risk or the presence of significant comorbidities. With the ageing population and increasing life expectancy, the prevalence of RAP is increasing, placing a substantial burden on healthcare systems and significantly reducing patients‘ quality of life. In recent decades, spinal cord stimulation (SCS) has emerged as a minimally invasive therapeutic option for the treatment of chronic pain of neuropathic origin, and its potential application in RAP has been increasingly explored [2]. SCS involves delivering low-intensity electrical pulses to the spinal cord at the T1–T2 vertebral region, aiming to modulate pain signals and influence vascular regulatory mechanisms. This innovative approach has shown promise in providing symptomatic relief and improving the quality of life for patients suffering from persistent angina pain, particularly when conventional interventions have failed.

Initial studies suggest that SCS can significantly improve symptoms, reduce the frequency of anginal episodes, decrease the reliance on anti-anginal medications, and enhance patients‘ quality of life [3]. However, the exact mechanisms by which SCS exerts its effects in RAP remain only partially understood. Proposed mechanisms include modulation of pain transmission via the ‘pain gate control’ theory, a reduction in sympathetic tone, and the redistribution of myocardial blood flow to ischaemic regions [4]. While early findings are encouraging, the clinical data available are still limited, and existing studies vary in design and methodology. This underscores the need for a critical and systematic analysis of the current evidence to better understand its therapeutic potential.

This review aims to provide a comprehensive summary of the current evidence supporting the use of SCS for RAP, exploring the clinical outcomes, hypothesized mechanisms, and future directions for this promising therapeutic approach.

## 2. Materials and Methods

This review was conducted through a systematic literature search on SCS for the treatment of RAP, aiming to analyze the available clinical evidence and provide a comprehensive, up-to-date overview of its efficacy, mechanisms of action, and safety profile.

### 2.1. Search Strategy and Study Selection

A search of major biomedical databases, including PubMed, Scopus, and Web of Science, was performed to identify articles published between 1990 and 2023. Specific search terms and keyword combinations, such as “spinal cord stimulation”, “refractory angina”, “chronic angina”, “neuromodulation”, and “pain management in angina”, were utilized. The search was restricted to studies published in English.

**Figure 1:**
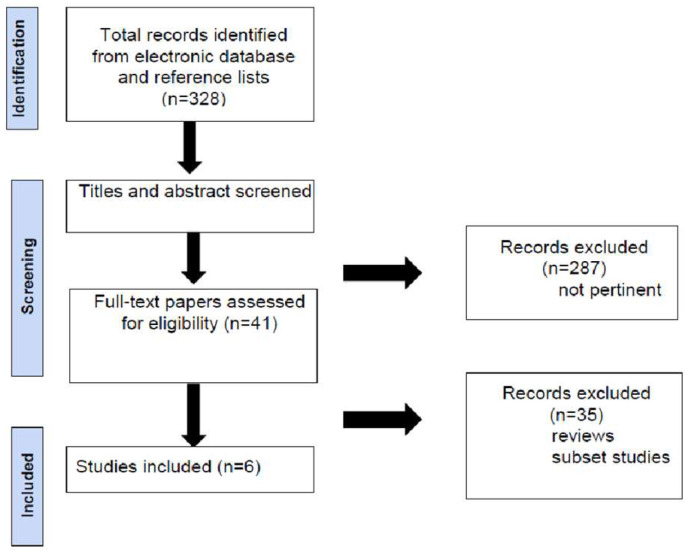
Flowchart of study selection process for SCS research.

A total of 328 articles were initially identified through the keyword search. After a review of the titles and abstracts, 287 articles were excluded as they did not meet the inclusion criteria regarding topic or study type. The remaining 41 articles were thoroughly reviewed in full to assess their relevance (Figure 1).

### 2.2. Inclusion and Exclusion Criteria

Included in the review: Randomised controlled clinical trials, observational studies, meta-analyses, and systematic reviews evaluating the efficacy of SCS in patients with RAP; studies with sample sizes larger than 10 patients to ensure statistical reliability and robustness.

Excluded in the review: Studies focusing on other applications of SCS (e.g., non-cardiac neuropathic pain); case reports and studies with small sample sizes (<10 patients); duplicate articles and unpublished studies in English.

After applying the inclusion and exclusion criteria, a total of six articles were selected for final analysis.

### 2.3. Data Extraction and Analysis

For each of the six included articles, detailed data were extracted on the following aspects: Study design and sample size; efficacy parameters, including reduction in angina episode frequency, nitroglycerin use, exercise capacity, and quality of life scores; safety profile, including the incidence of complications

## 3. Summary of Evidence

The results of the selected studies were organized to provide a comprehensive overview of the use of SCS in RAP. Key findings highlighted clinical outcomes in terms of symptom reduction and improvement in quality of life among patients undergoing SCS treatment. Additionally, the hypothesized mechanisms of action, including modulation of pain signals, autonomic balance improvement, and myocardial perfusion redistribution, were explored and interpreted in the context of the clinical data. A comparison of stimulation protocols, such as paresthetic versus subperception stimulation, revealed varying impacts on angina pain, underscoring the importance of tailoring treatment approaches to individual patient needs. This analysis not only identified emerging clinical trends but also uncovered significant gaps in the current literature, emphasizing the necessity for further research to refine and optimize the role of SCS in the management of refractory angina.

### 3.1. Mechanism of Action of SCS

**Figure 2:**
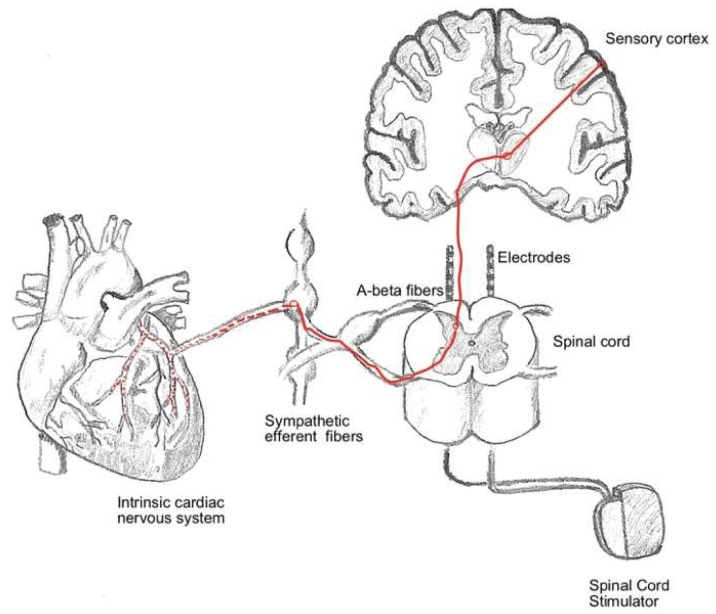
Mechanism of action of SCS in RAP.

The precise mechanism of action of SCS in RAP remains incompletely understood, but several interrelated theories have been proposed to explain its efficacy in reducing angina pain and enhancing quality of life. Central to these mechanisms is the modulation of pain signaling pathways. According to the gate control theory, SCS delivers low-intensity electrical pulses to the spinal cord, which interfere with nociceptive transmission by activating inhibitory interneurons in the dorsal horn. This process effectively “closes the gate” to pain signals ascending to the brain, thereby reducing the perception of anginal pain. Beyond pain modulation, SCS may have indirect effects on myocardial perfusion. By altering sympathetic and parasympathetic nervous system activity, SCS has been hypothesized to redistribute blood flow within the myocardium, preferentially directing it toward ischemic regions. This autonomic modulation may help alleviate myocardial ischemia, contributing to symptomatic relief. Additionally, SCS has been shown to reduce myocardial oxygen demand, which could further mitigate ischemic pain. Another proposed mechanism involves the improvement of autonomic balance. SCS may reduce sympathetic overactivity, which is often heightened in patients with refractory angina, and enhance parasympathetic tone. This shift in autonomic regulation can potentially lower the overall stress response on the cardiovascular system, resulting in improved outcomes. These mechanisms highlight the multifaceted impact of SCS on both sensory perception and physiological processes underlying anginal symptoms. Figure 2 illustrates the interplay of these mechanisms, providing a visual summary of the hypothesized process.

In the subsequent subsections, a comprehensive exploration of the various mechanisms underlying SCS in RAP is presented. Each mechanism is discussed in detail, providing an in-depth understanding of how SCS exerts its therapeutic effects. These subsections delve into the intricate processes of pain modulation, myocardial perfusion redistribution, and autonomic nervous system regulation, all of which contribute to the observed clinical benefits of SCS. 

#### 3.1.1. Modification of Pain Transmission: Gate Control Theory

The ‘gate control theory’, first introduced by Melzack and Wall, is a fundamental concept in understanding the analgesic effects of SCS [5]. According to this theory, the stimulation of non-nociceptive (Aβ) nerve fibers in the spinal cord inhibits the transmission of pain signals from nociceptive (Aδ and C) fibers. This process acts as a “gate control”, selectively blocking pain signals from reaching higher brain centers, thereby modulating pain perception. In the context of RAP, SCS is believed to alleviate chest pain by interfering with the nociceptive signaling pathways at the spinal level. By effectively “filtering” these pain signals, SCS can directly reduce the perception of angina pain, leading to a decrease in the severity and frequency of chest pain episodes, ultimately improving the patient’s quality of life.

#### 3.1.2. Reducing Sympathetic Tone and Improving Autonomic Balance

SCS may also exert its effects through modulation of the autonomic nervous system, promoting a reduction in sympathetic tone and achieving a better balance between the sympathetic and parasympathetic systems [6]. By decreasing sympathetic activity, SCS can lower myocardial oxygen demand, thereby alleviating ischaemia. This mechanism not only improves the oxygenation of myocardial tissue but also helps stabilize the cardiovascular system, potentially reducing the risk of ischaemic events and arrhythmias commonly associated with RAP. The autonomic modulation provided by SCS thus represents a significant mechanism contributing to both symptomatic relief and enhanced quality of life in patients undergoing this therapy.

#### 3.1.3. Myocardial Flow Redistribution: The ‘Robin Hood’ Effect

Another hypothesis suggests that SCS may enhance perfusion in ischaemic regions of the myocardium through a redistribution of blood flow, a phenomenon commonly referred to as the ‘Robin Hood effect’ [7]. This mechanism implies that SCS preferentially directs blood flow to the most ischaemic areas of the heart by reducing perfusion in non-ischaemic regions. This redistribution could be mediated by parasympathetic-induced vasodilation or by a reduction in perfusion pressure within healthy vessels, thereby allowing a greater supply of blood to oxygen-deprived areas. Such an effect on myocardial perfusion may contribute significantly to the observed symptom relief and improved clinical outcomes in patients with RAP undergoing SCS therapy.

#### 3.1.4. Potential Effects on Endothelial Function and Microvasculature

Recent imaging studies indicate that SCS may enhance endothelial function and promote the development of vascular collaterals in ischaemic regions [8]. This mechanism could provide a sustained improvement in myocardial perfusion, functioning independently of its pain-modulating effects. Although the precise processes remain unclear, emerging evidence suggests that SCS may facilitate the release of endothelial mediators, such as nitric oxide, which enhance vasodilation and vascular health. Additionally, the formation of vascular collaterals could improve blood supply to deprived myocardial areas, exerting a protective and potentially regenerative impact on coronary microcirculation.

### 3.2. Surgical Procedure of SCS

In general, the implantation of an SCS system involves two distinct phases: the trial phase and the permanent implantation phase. Patients who respond positively during the trial are good candidates for permanent implantation of the SCS.

The Trial Phase: this initial phase is conducted to evaluate the effectiveness of SCS in relieving pain. The procedure is performed on lightly sedated patients in the prone position. After administering local anaesthesia to the skin and subcutaneous tissues, a small linear skin incision is made between the L2 and L5 vertebrae. Under real-time X-ray guidance, a Tuohy needle is advanced into the epidural space. An electrode is then introduced through the needle and guided upwards into the epidural space to the targeted level, typically at T1–T2 for RA treatment. Intraoperative mapping is used to confirm that the paraesthesia induced by the electrode adequately covers the painful areas. Once the electrodes are correctly positioned, they are anchored to the lumbar fascia. The extension cables are then connected to an external pulse generator (EPG) for temporary use during the trial period. Patients who report significant pain relief during this phase are considered suitable candidates for permanent implantation.

The Permanent Implantation Phase: if the trial phase is successful, the extension cables are removed, and the permanent electrodes are tunneled to a subcutaneous pocket, typically created in the upper part of the buttock. In this pocket, the internal pulse generator (IPG), which serves as the battery and control unit, is implanted and connected to the electrodes. 

### 3.3. Patient Selection

Patient selection is a crucial step in optimizing the effectiveness of SCS for RAP while minimizing the risk of complications. RAP is a challenging condition predominantly seen in patients with advanced coronary artery disease, for whom conventional therapies—whether pharmacological or interventional—are either ineffective or contraindicated. As a result, SCS is specifically reserved for a well-defined subset of patients who meet stringent clinical criteria to ensure the best possible outcomes. 

#### 3.3.1. Inclusion Criteria

Candidate patients for SCS must present with:

- Documented RAP resistant to pharmacological treatment and unresponsive to surgical or percutaneous revascularization options.
- Absence of surgical contraindications for device implantation.
- Motivation and adherence to treatment. Device management requires regular follow-up, as well as an understanding of its limitations and realistic expectations. Patients must demonstrate the ability to manage the device effectively and have an adequate understanding of the treatment protocol.

#### 3.3.2. Exclusion Criteria

Exclusion criteria for SCS include:

- Active infections or high risk of infection: the presence of a pulse generator and electrodes can increase susceptibility to infection.
- Significant psychological problems or cognitive disorders that could impair the patient‘s ability to manage the device or follow the treatment plan.
- Presence of incompatible implantable pacemakers or defibrillators, which could interfere with the functionality of the SCS.
- Severe haemodynamic instability.

#### 3.3.3. Pre-Operative Assessment

Prior to SCS implantation, patients should undergo a comprehensive evaluation, which includes: cardiological evaluation and coronarography to confirm the absence of alternative revascularization options; imaging examinations (spinal MR or CT) to accurately plan electrode placement; psychological screening: to assess the patient’s mental readiness and suitability for managing the device and adhering to treatment.

### 3.4. Spinal Cord Stimulation and RAP

SCS initially developed for the treatment of neuropathic pain, has demonstrated effectiveness in alleviating RAP symptoms and enhancing the quality of life for patients who lack other therapeutic options. There is considerable opposition among cardiologists to the use of SCS for RAP. The primary concern is that SCS may mask or reduce precordial pain associated with myocardial infarction, potentially eliminating this critical warning sign and posing a safety risk. However, in RAP, SCS relieves pain by reducing the transmission of nociceptive impulses through the spinothalamic tract. Additionally, SCS exerts a direct anti-ischemic effect by inducing vasodilation through the release of calcitonin gene-related peptides and reducing myocardial oxygen demand via lowered sympathetic tone. Furthermore, the imbalance between oxygen supply and demand that causes ischemia can be mitigated by improving coronary blood flow at the microvascular level, resulting in subsequent pain relief. Moreover, SCS improves ischemic conditions by decreasing the incidence of arrhythmias or ischemia through the inhibition of excessive intrinsic cardiac nervous system activity, reducing sympathetic activity, and redistributing myocardial blood flow from non-ischemic to ischemic areas [4]. Effective collaboration between cardiologists and physicians specializing in SCS implantation is essential to ensure the proper selection of candidates who would benefit most from SCS, optimizing patient outcomes and minimizing risks.

The following paragraphs will delve into the evidence of clinical efficacy, explore the impact of SCS on quality of life, and provide a comparative analysis with other treatment modalities for RAP, highlighting the unique benefits and limitations of SCS in this challenging patient population.

#### 3.4.1. Evidence of Clinical Efficacy

Several studies have demonstrated the efficacy of SCS in managing RAP. In a study by Mannheimer et al. [9], SCS was compared to coronary artery bypass grafting (CABG) in high-surgical-risk patients. The results showed that patients treated with SCS experienced reductions in anginal pain and nitroglycerin use comparable to those achieved with CABG but with a significantly lower six-month mortality rate (1.9% in the SCS group versus 13.7% in the CABG group) and fewer cerebrovascular events (3.8% versus 15.7%). The ESBY study conducted by Norrsell et al. in 2000 [10] further corroborated the benefits of SCS in managing refractory angina. This randomized trial compared SCS with CABG in patients with severe angina. Both groups demonstrated significant reductions in anginal episodes and nitroglycerin use. However, while CABG was associated with a greater decrease in ischemic burden and ST-segment changes, the SCS group showed fewer complications and a similar overall improvement in quality of life. Notably, in the SCS group, many patients continued to experience relief from anginal symptoms even after the spinal cord stimulation was discontinued, suggesting a potential lasting analgesic or neuroplastic effect. These findings highlight the potential of SCS as a less invasive alternative for patients unable or unwilling to undergo surgical revascularization [10]. Moreover, in 2011 the SCS-ITA Trial compared paresthesia stimulation (perceived) with subliminal stimulation (not perceived) and placebo stimulation (‘sham’) in a cohort of patients with RAP who underwent SCS implantation [11]. The group receiving paresthesia stimulation showed a significant reduction in angina attacks and nitroglycerin consumption compared to the placebo group, suggesting that the perception of stimulation may play a key role in the therapeutic effectiveness of SCS. Further evidence of SCS efficacy in RAP was provided by a meta-analysis conducted by Pan et al. in 2017 [2], which included 12 randomized trials with a total of 476 patients. The analysis revealed significant reductions in the frequency of angina episodes and nitroglycerin use in patients treated with SCS, along with improvements in exercise time and subjective pain perception. However, the impact of SCS on exercise tolerance was not always statistically significant, indicating that its benefits may be more pronounced for alleviating subjective symptoms rather than enhancing physical performance.

#### 3.4.2. Spinal Cord Stimulation and Quality of Life

In addition to reducing pain, SCS has been shown to significantly improve the quality of life in patients with RAP. A long-term follow-up study by Vervaat et al. [12] monitored 132 patients over an average period of 65 months, demonstrating that SCS not only decreased the frequency of anginal episodes but also enhanced quality-of-life scores, as assessed by the Seattle Angina Questionnaire (SAQ). The frequency of anginal episodes was significantly reduced, with most patients reporting nitroglycerin use less than once a week. Moreover, the long-term safety of SCS was supported by a low complication rate, with fewer than 3% of patients requiring device removal due to infection or positioning issues.

#### 3.4.3. Comparison with Other Treatments for RAP

A comparison of SCS with other therapeutic modalities highlights its advantages for specific patient profiles. The SPiRiT Trial compared SCS with transmyocardial laser revascularization (TMR) in patients with RAP, finding that both therapies provided comparable benefits in symptom reduction [13]. However, TMR is a more invasive procedure associated with a higher risk of complications, positioning SCS as the preferred option for patients who are unable to tolerate complex surgical interventions.

An overview of the variability of outcomes in SCS treatment in refractory angina is reported in Table 1.

### 3.5. New Modalities of Neurostimulation and Refractory Angina

SCS technology has advanced significantly in recent years, resulting in the development of novel neurostimulation modalities that have broadened the therapeutic options for patients with refractory conditions like angina [14]. These modalities include high-frequency stimulation (10 kHz), burst stimulation, and differential target multiplexed (DTM) stimulation. Each has demonstrated efficacy in managing chronic pain without requiring paresthesia mapping, thereby simplifying the treatment process for patients and improving overall accessibility and comfort. Unfortunately, the evidence supporting these new SCS paradigms in the context of RAP remains limited.

#### 3.5.1. High-Frequency Stimulation

High-frequency stimulation (10 kHz) delivers high-frequency pulses with low amplitude, enabling subthreshold stimulation without inducing paresthesias [15]. By eliminating the reliance on paresthesias, this approach facilitates easier anatomical placement of electrodes and enhances patient comfort. Kapural et al. [15] demonstrated that 10 kHz stimulation significantly reduces pain scores in patients with chronic pain compared to conventional stimulation. In the context of refractory angina, this modality has the potential to reduce the discomfort associated with paresthesias, thereby improving both tolerability and adherence to therapy.

#### 3.5.2. Burst Stimulation

Burst mode, developed by De Ridder, utilizes intermittent pulse frequencies interspersed with rest phases between stimulation cycles [16]. Studies have demonstrated that Burst stimulation outperforms traditional stimulation in managing chronic pain, offering significant reductions in long-term pain scores and improved patient tolerability [17]. This stimulation approach modulates pain signals in a manner that more closely mimics natural neural patterns, potentially enhancing pain management effectiveness for patients with RA. However, to date, no comprehensive studies beyond case reports and an ongoing trial have been conducted on the use of burst stimulation for the treatment of RAP [18,19].

#### 3.5.3. Fast Stimulation

Fast-Acting Sub-Perception Therapy (FAST) employs sub-perception stimulation combined with paresthesia-based low-frequency stimulation, utilizing multiple waveforms simultaneously. This approach allows for the customization of neurostimulation patterns to meet individual patient needs.

#### 3.5.4. Differential Multiplex Stimulation (DTM)

DTM is an advanced neurostimulation technology designed to target not only neurons but also glial cells, which play a critical role in neural support and modulation [20]. By influencing processes such as inflammation and cellular regulation, DTM alters the gene expression of glial cells in the dorsal horn, thereby modulating the activity of pain-associated neurons. Preliminary studies have demonstrated that DTM may provide more sustained pain relief compared to traditional stimulation techniques, making it a promising therapeutic option for patients with RAP [21]. These innovative neurostimulation modalities represent a significant advancement in the management of RAP. They offer the potential to enhance treatment efficacy, improve patient quality of life, and reduce side effects associated with paresthesia, thereby addressing some of the limitations of conventional neurostimulation therapies.

### 3.6. Discussing the Need for Trials in the Treatment of Rap

Currently, the value of trials in pain treatment is debated, as many potential responders are denied long-term treatment due to false-negative trial results. However, in the context of RAP, the “direct-to-implant” approach is considered the standard approach [22,23]. Typically lasting one or more weeks, the trial phase serves to evaluate the patient‘s response to therapy, optimize electrode positioning, and reduce the risk of definitive treatment failure. However, the costs and logistical complexities associated with this phase have led some centers to explore a “direct-to-implant” or “all-in-one” approach, bypassing the trial phase to streamline the process and conserve resources. The direct-to-implant strategy is currently the standard approach for RAP cases, with established safety and efficacy. Economically, the trial phase can reduce long-term costs by identifying non-responders early, thus avoiding unnecessary permanent implantations and associated expenditures. However, in RAP patients, the trial phase may significantly increase surgical and anesthesia risks without offering additional benefits. Notably, recent studies reporting a two-stage approach in RAP patients remain limited [24,25].

In conclusion, the trial phase can provide valuable insights only in certain highly selected cases, and bypassing the trial phase is generally recommended for patients with RAP. The direct-to-implant approach simplifies the process, reduces procedural risks, and aligns with current evidence supporting its safety and efficacy in RAP. Balancing clinical outcomes with cost-effectiveness will remain crucial in optimizing patient selection and treatment strategies.

### 3.7. Complications

Despite the established efficacy of SCS in treating RAP, the procedure carries inherent risks and potential complications, both during the implantation phase and throughout long-term use. Understanding these complications is essential for ensuring appropriate patient selection, optimizing postoperative care, and maintaining effective long-term monitoring.

#### 3.7.1. Perioperative Complications

Perioperative complications are infrequent but can include the following:

- Infections: infection at the site of the pulse generator or epidural electrodes is among the most common complications, potentially requiring device removal. When looking at all indications for SCS, the reported infection rates range from 3% to 8%, with a higher incidence in immunocompromised patients or those with comorbidities such as diabetes [26]. In patients with RAP, the reported rates of adverse events are 1% for infections and 7.8% for device-related [3]. In their series of 132 RAP patients, Vervaat et al. [27] reported two cases (1.5%) of SCS-related infections: in one patient this resulted in the removal of the SCS, while in the other, a superficial infection of the IPG pocket was successfully treated with antibiotics.
- Epidural haematoma: although rare, an epidural hematoma is a serious complication that can result in spinal cord compression and neurological deficits. Prompt intervention and meticulous surgical technique are critical in mitigating this risk [28]. Although the risk of bleeding in RAP patients undergoing invasive treatments is increased due to antithrombotic therapy, there are no reported cases of epidural hematoma associated with SCS implantation.
- Electrode Displacement: displacement or malpositioning of electrodes can undermine the effectiveness of treatment, often necessitating surgical revision. Electrode displacement typically occurs during or shortly after the procedure, with an incidence of 5% to 10% within the first few months post-implantation [29].

#### 3.7.2. Long-Term Complications

Long-term complications can arise months or even years after SCS implantation, including:

- IPG malfunction: The functionality and battery life of the IPG are critical for sustained therapy. Malfunction or the need for battery replacement is a common reason for surgical revision. Advances in rechargeable battery technology are helping to reduce this issue [30].
- Paresthesias or Unpleasant Sensations: While paresthetic stimulation is a typical feature of SCS, some patients experience unpleasant or painful sensations that may compromise adherence to therapy. Emerging modalities, such as high-frequency stimulation, are being investigated to minimize these sensations and improve patient comfort.
- Tolerance phenomenon: Over time, some patients develop tolerance to SCS, resulting in diminished efficacy in pain control. This tolerance often necessitates frequent adjustments to device settings and, in some cases, reconsideration or revision of the treatment plan [31].

### 3.8. Interferences and Interactions with Other Medical Devices

The presence of implantable devices, such as pacemakers or cardiac defibrillators, can interfere with SCS functionality, potentially reducing its efficacy or posing risks to the patient. Interactions between SCS and other implantable devices must be carefully assessed and monitored, particularly in patients with cardiovascular comorbidities requiring multiple devices. Clinicians must account for the presence of such devices during patient selection and ensure vigilant follow-up to mitigate any potential complications or adverse interactions. Although interference between cardiac devices and SCS systems is a rare occurrence and no specific guidelines currently exist, it is recommended that cardiac devices be programmed to their maximum sensitivity to mitigate any potential interference from SCS. [32] This precaution helps ensure the safety and functionality of both devices in patients requiring dual implantation.

### 3.9. Management of Complications

The management of complications necessitates careful follow-up and a multidisciplinary approach. In cases of superficial surgical site infections, prompt treatment with antibiotics can often prevent the need for device removal. However, deep infections involving the implant typically require device removal to ensure complete resolution [33]. However, complications such as electrode dislocation or IPG malfunction frequently require surgical intervention. The adoption of advanced technologies, including rechargeable devices and innovative stimulation modalities, holds promise for reducing long-term complication rates and enhancing patient quality of life.

## 4. Future Perspectives

The future of SCS in treating RAP appears promising, driven by technological advancements and increasing clinical evidence. However, further research is essential to deepen the understanding of its mechanisms of action, optimize stimulation modalities, and refine patient selection criteria to enhance both the efficacy and safety of this treatment.

### 4.1. Innovations in Stimulation Modalities

In recent years, new SCS modalities, including high-frequency stimulation, Burst, and DTM, have demonstrated potential not only in the treatment of neuropathic pain but also in addressing other refractory conditions such as RAP [18,19]. These paresthesia-free technologies offer the promise of enhanced patient comfort and tolerability while reducing the risk of tolerance development. Future research should focus on evaluating the efficacy of these innovative modalities for RAP pain management, comparing their outcomes with traditional stimulation methods, and optimizing parameters to tailor treatment to individual patient needs.

### 4.2. Personalized Approach and Predictive Biomarkers

Another crucial area of development is the implementation of a personalized approach to SCS, tailored to individual patient characteristics. The identification of predictive biomarkers has the potential to enhance candidate selection, thereby increasing the likelihood of therapeutic success. For instance, analyzing neurophysiological responses, genetic profiles, and specific clinical characteristics could enable clinicians to pinpoint patients who are most likely to respond favourably to SCS. This precision-based strategy could significantly reduce treatment failures, optimize therapeutic outcomes, and improve overall efficiency in the management of refractory conditions.

### 4.3. High-Quality Clinical Trials and New Study Designs

Despite the growing evidence supporting the use of SCS for RAP, many studies are limited by small sample sizes and the absence of placebo controls. To strengthen the evidence base, it is crucial to conduct large, multicenter, randomized controlled trials with placebo groups to accurately compare the efficacy of SCS against other treatment options. Additionally, direct-to-implant trials, which omit the preliminary trial phase, may offer a viable alternative for selected patient populations. However, further research is needed to validate the safety and efficacy of this approach before it can be widely adopted.

### 4.4. Development of Intelligent and Customized Devices

Technological advancements are creating exciting opportunities to enhance the management and efficacy of SCS. Next-generation stimulation devices may incorporate remote monitoring capabilities and artificial intelligence (AI) algorithms that dynamically adjust stimulation parameters based on real-time patient feedback. These “smart” systems could continuously monitor physiological data, automatically optimizing stimulation to maintain therapeutic efficacy and reduce side effects. Such innovations have the potential to improve treatment outcomes, enhance patient adherence, and increase overall satisfaction with the therapy.

### 4.5. New Frontiers in Mechanisms of Action Research

A pivotal area for future research lies in deepening the understanding of the mechanisms of action underlying SCS. Advanced physiological investigations, including brain imaging techniques and analyses of autonomic nervous system responses, hold the potential to clarify how SCS alleviates anginal pain and enhances myocardial perfusion. Gaining a more comprehensive understanding of these mechanisms could not only optimize SCS therapy for angina but also broaden its application to other cardiac conditions, thereby unveiling new possibilities for neuromodulation in cardiovascular care.

### 4.6. Closed Loop Devices and Real-Time Stimulation Control

One of the most recent advancements in SCS technology is the development of closed-loop devices [34]. These systems are designed to monitor the patient’s physiological responses in real-time, such as variations in spinal cord electrical signals, and automatically adjust stimulation intensity and parameters based on the detected data. Unlike traditional open-loop devices, which deliver constant, pre-set stimulation, closed-loop systems dynamically adapt the stimulation to maintain consistent efficacy and minimize fluctuations in treatment response.

This innovative technology has the potential to significantly enhance the efficacy of SCS in managing RAP, reducing the incidence of side effects such as paresthesias and minimizing the development of tolerance. By enabling a more personalized and responsive approach to stimulation, closed-loop devices represent a major step forward in tailoring therapy to the patient’s immediate needs. Future studies should focus on evaluating the efficacy and safety of closed-loop systems specifically for RAP, exploring their impact on symptom control, quality of life, and patient adherence to treatment protocols.

## 5. Conclusions

SCS represents a groundbreaking and promising therapeutic option for patients with RAP, a group often left with limited treatment alternatives due to the ineffectiveness or impracticality of conventional approaches. Evidence demonstrates that SCS can significantly alleviate anginal pain and enhance quality of life, establishing it as a viable alternative for patients unresponsive to pharmacological and interventional therapies. Recent advancements in SCS technology, such as high-frequency stimulation, burst, DTM, and closed-loop systems, offer new opportunities to optimize treatment. These innovations aim to improve patient tolerability, reduce the phenomenon of tolerance, and provide more consistent therapeutic outcomes. While these modalities have shown success in managing chronic pain, further research is necessary to evaluate their specific efficacy in the context of RAP. Looking forward, a more personalized approach to SCS is anticipated, with the identification of predictive biomarkers to refine patient selection and increase the likelihood of therapeutic success. Additionally, the integration of cutting-edge technologies, such as remote monitoring and AI-driven systems, could enhance the efficiency of treatment by enabling real-time adjustments, improving patient adherence, and boosting long-term effectiveness. Despite its potential, challenges remain. 

Historically, cardiologists have been reluctant to adopt SCS as part of their treatment armamentarium or to involve interventional pain specialists and neurosurgeons in the management of patients with RAP. This reluctance is often attributed to the lack of regulatory approvals and large-scale studies, despite nearly three decades of clinical experience. Establishing strong collaboration between neurosurgeons, interventional pain specialists, and the cardiology community is essential to integrate SCS into cardiology treatment algorithms for RAP effectively. High-quality, large-scale clinical trials with placebo-controlled designs are essential to solidify the evidence base for SCS in RAP. Furthermore, the utility of the trial phase continues to be debated, necessitating further studies to determine whether this step can be bypassed under certain conditions without compromising outcomes. In conclusion, SCS has proven to be an effective and relatively safe therapy for patients with RAP, offering hope to those who have exhausted conventional treatment options. With ongoing technological and clinical advancements, SCS has the potential to become a cornerstone in the management of this complex condition, significantly improving the quality of life for a challenging patient population.

## Figures and Tables

**Table 1:** Overview of the variability of outcomes in SCS treatment in refractory angina.

References	N° pts	Study Characteristics	SCS Paradigm	Follow Up	Outcomes
Mannheimer et al., 1998 [9]	104	Randomized	Tonic	6 months	lower mortality rate (*p* = 0.02) lower cerebrovascular morbidity (*p* = 0.03)
Norrsell et al., 2000 [10]	53 (SCS), 51 (CABG)	Randomized	Tonic	12 months	number and duration of ischemic episodes (CABG vs. SCS both *p* < 0.05) angina attacks (preoperative vs. follow-up *p* < 0.0001) both CABG and SCS.
McNab et al., 2006 [14]	68	SPiRiT trial, vs. PMR	Tonic	12 months	CCS reduction >2 classes in 37% at 3 and 12 months.
Lanza et al., 2011 [11]	25	randomized	subliminal vs. tonic	3 months	reduction anginal episodes (SCS vs. placebo *p* < 0.016) Nitroglycerin use (decreased in paresthetic SCS group (*p* < 0.015) SAQ scores in stability and frequency (*p* = 0.009 and *p* = 0.008), respectively. CCS class improvement from 3.30 to 2.10 in paresthetic SCS group. No difference compared to placebo in subliminal SCS group.
Pan et al., 2017 [2]	476	Meta analysis	Tonic and high frequency	variable	Exercise time increased in SCS group (*p* = 0.008) CCS class changes improve (*p* = 0.01) VAS pain score reduction (*p* = 0.001) angina frequency significant decrease (*p* = 0.008) nitroglycerin use significant reduction (*p* < 0.00001) significant reduction in SAQ (*p* = 0.007)
Vervaat et al., 2023 [12]	132	Cohort prospective	tonic	65 months	SAQ improvement (*p* < 0.001) Angina episodes reduction (*p* < 0.001) nitroglycerin use reduction (*p* < 0.001) RAND-36 improvements (*p* = 0.001)

## Data Availability

Not applicable.
